# The impact of surgical learning curve on survival – reopening the door for minimally invasive surgery in the management of cervical cancer?

**DOI:** 10.1111/1471-0528.16485

**Published:** 2020-10-07

**Authors:** H Falconer

**Affiliations:** ^1^ Department of Pelvic Cancer Karolinska University Hospital Stockholm Sweden; ^2^ Department of Women's and Children's Health Karolinska Institutet Stockholm Sweden

Minimally invasive surgery has been widely adopted in gynaecological oncology with a significant surge since 2010 associated with the introduction of robot‐assisted surgery. Up until 2018, virtually all testimonials of minimally invasive surgery for cervical cancer indicated equal oncological outcomes compared with laparotomy. The unexpected results from the only randomised controlled trial (Ramirez et al. *N Engl J Med* 2018;379:1895–904) seriously challenge the use of minimally invasive surgery for cervical cancer. However, the lack of plausible explanations for the inferior survival has made it hard for many gynaecological oncologists to accept the results at face value. Suggested causes such as the use of intrauterine manipulators and intracorporeal colpotomy may to some extent account for the detrimental outcomes but as practice differs widely worldwide, none of these factors seem convincing. In the current paper by Baeten et al., a different aspect of novel surgical technologies has been explored (*BJOG* 2020; https://doi.org/10.1111/1471-0528.16399). The adoption of any new modality/technology is clearly associated with a learning curve and it is well known from surgery that procedural outcomes, such as operative time, improve with increasing case numbers. However, the potential impact of learning curve on hard outcomes such as survival has scarcely been reported. Baeten et al. convincingly demonstrate that a substantial number of robot‐assisted radical hysterectomies are required to overcome an initial harm. The authors point out that the learning curve should be considered institutional; suggesting that an individual learning curve may benefit from previous experiences at the specific institution. Intriguingly, similar data have not been presented for endometrial cancer, which may be related to the higher complexity of radical hysterectomy for cervical cancer. Although no specific changes were made over time in the current study, a learning curve may include modifications of surgical routines based on early experiences. Which specific improvements during the learning curve account for the increased survival are unclear but reduction of complications that could delay adjuvant treatment may be one. The data presented by Baeten et al. signify a solid effort to elucidate the underlying cause of the outcomes from the LACC trial, especially as the trial was launched at a time when most surgeons had limited experience from laparoscopic/robotic radical hysterectomy. The study should raise the awareness of surgical learning curve in the context of survival in oncological surgery. It also brings us to the most important question – how do we avoid harming our patients when novel technologies are adopted? The authors suggest that centralisation and structured training represent two of the most important strategies to mitigate the effects of early errors. Interestingly, recent population‐based studies from countries with high levels of centralisation do not corroborate the findings from the LACC trial (Alfonzo et al. *Eur J Cancer* 2019;29:1072–6; Jensen et al. *Eur J Cancer* 2020;128:47–56). Although the current study may reopen the door for robotic surgery in the management of cervical cancer, prospective randomised trials are needed to ensure its safety. The impact of surgical learning curve should be considered in any future trial exploring oncological safety for procedural interventions.

## Disclosure of interests

HF is a proctor for Intuitive Surgical Inc. A completed disclosure of interests form is available to view online as supporting information.

## Supporting information

Supplementary Material

